# Comparative Analysis of Polyurethane Drive Belts with Different Cross-Section Using Thermomechanical Tests for Modeling the Hot Plate Welding Process

**DOI:** 10.3390/ma14143826

**Published:** 2021-07-08

**Authors:** Krzysztof Wałęsa, Anna Wrzesińska, Marta Dobrosielska, Krzysztof Talaśka, Dominik Wilczyński

**Affiliations:** 1 Institute of Machine Design, Faculty of Mechanical Engineering, Poznan University of Technology, Piotrowo 3 Street, 61-138 Poznań, Poland; krzysztof.talaska@put.poznan.pl (K.T.); dominik.wilczynski@put.poznan.pl (D.W.); 2 Centre for Advanced Technologies, Adam Mickiewicz University in Poznan, Uniwersytetu Poznańskiego Street 10, 61-614 Poznań, Poland; annwrz1@amu.edu.pl; 3 Faculty of Materials Science and Engineering, Warsaw University of Technology, Wołoska Street 141, 02-507 Warszawa, Poland; m.dobrosielska0000@gmail.com

**Keywords:** hot plate welding, thermoplastic elastomers, material analysis, round belts welding, polyurethane belts, thermomechanical test, DSC analysis, DMTA analysis, specific heat, glass transition temperature

## Abstract

The paper presents a comparative analysis of the circular and flat cross-section belts using measurements of a set of thermomechanical parameters, contributing to research about hot plate welding of drive belts. On the basis of thermogravimetric and spectrophotometric tests, information about the same chemical composition of the two belts was obtained. Dynamic thermomechanical analysis and scanning differential calorimetry provided information about a small difference between belts, which disappeared when the material was placed in a state of increased temperature and mechanical stress. On the basis of the analysis of the specific heat, thermal diffusion, density, and hardness, the values of the selected thermal properties of the belt were obtained, and a large similarity between the belts was identified. On the basis of the novel performed test cycle, it has been hypothesized that circular and flat belts made from thermoplastic polyurethane elastomer could be used interchangeably for butt-welding testing. It has also been proven that cyclic thermomechanical loads unify the properties of both materials so that multiple mechanical and thermal loads do not result in any change in the material properties of the two belts. As a consequence, changes in the weld properties after welding, compared to a solid belt, are not expected.

## 1. Introduction

### 1.1. Origins of the Research Problem

Drive belts with a circular cross-section and a diameter of several millimeters are commonly used in the drive and transport systems of industrial machinery [1,2]. In many cases, they are made from thermoplastic elastomers, in particular polyurethane and polyester [3,4]. The production process is two-step. In the first step, a belt length of several hundred meters is produced by continuous extrusion. This stage ends with winding the belt onto the spool, which facilitates transport and storage [5]. The final stage of the production of a finished drive or conveyor belt consists of trimming the belt to an appropriate length and making a permanent connection between its ends. This results in a drive belt with a specified circumference length [5,6].

In many cases, the belt ends are connected by the hot plate butt welding. This method is known as a cheap and easy way to combine thermoplastic materials [7]. This process is usually done manually, using dedicated, simple tools. Unfortunately, this action does not provide satisfactory repeatability of the geometric dimensions of the belt after welding, as well as high quality of welding. This is due to the manual application of the force needed to plasticize and connect the belt ends, which does not ensure the repeatability of the conditions of this process [3,8,9,10]. In response to the industry demand for automation of the hot plate butt welding of drive belts, the authors have proposed a solution for the automatic belt welding machine. The proper configuration of the control system requires the development and verification of the technological parameters of the welding process [3,11,12].

### 1.2. Hot Plate Welding Process

Description of the process of hot plate butt welding of drive belts provides information on physical phenomena occurring during the process. Their diagnosis is essential for the following:

- Developing a mathematical model of the welding process, which will be used to derive the relationship of welding parameters to the process input sizes (material type, belt diameter),
- Identification of the physical properties of the welded material, the knowledge of which is necessary to determine the technological parameters of the process [13,14].

**Figure 1:**
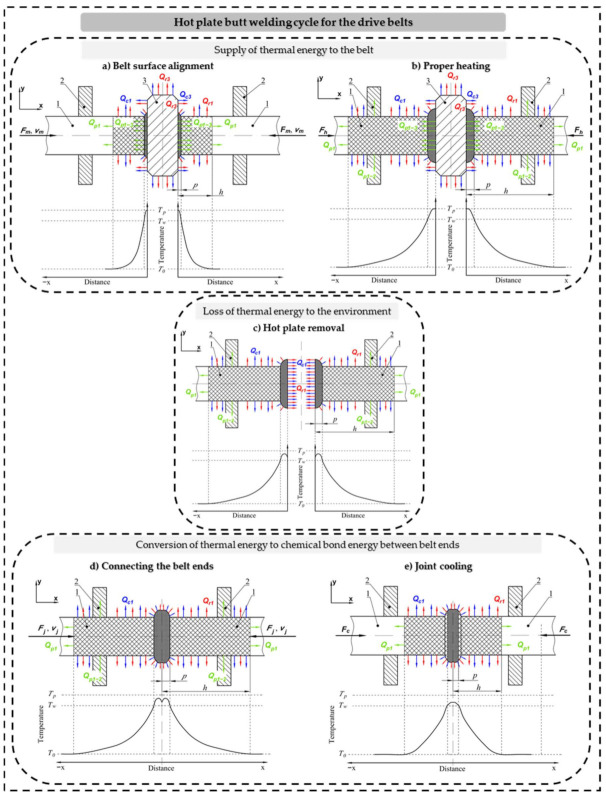
Hot plate butt welding cycle for the drive belts: (**a**) belt surface alignment, (**b**) proper heating, (**c**) hot plate removal, (**d**) connecting the belt ends, (**e**) joint cooling; 1—belt, 2—belt handle, 3—hot plate; *F_m_*—force aligning the belt on the hot plate, *F_h_*—force pressing the belt to the hot plate during heating, *F_j_*—force pressing the belt during joining, *F_c_*—pressing force during cooling of the joint, *v_m_*—velocity of pressing the belt ends to the hot plate during alignment, *v_j_—*velocity of pressing the belt during joining, *T_p_*—hot plate temperature, *T_w_*—welding temperature, *T*_0_—ambient temperature, *p*—belt plasticity distance, *h*—distance heated to temperature above ambient temperature; *Q_p_*_3-1_—heat conducted between the hot plate and the belt, *Q_p_*_1-2_—heat conducted between the belt and the handle, *Q_p_*_1_—heat conducted inside the belt material, *Q_r_*_1_—heat released from the belt to the ambient by radiation, *Q_r_*_3_—heat released from the hot plate to the ambient by radiation, *Q_c_*_1_—heat released from the belt to the ambient by convection, *Q_c_*_3_—heat released from the hot plate to the ambient by convection.

Taking into account the basic operations of the process of butt welding of drive belts, it can be divided into five stages [15,16] (Figure 1):

- Belt alignment on the surface of the hot plate (Figure 1a), during which the ends of the belt (1), captured by the jaws (2), are pre-pressed to the surface of the hot plate (3) at velocity *v_m_*. Upon reaching contact between the hot plate and the belt ends, they are pressed against it with force *F_m_*. This action is intended to pre-melt uneven parts of the belt head and adjust it to the surface of the hot plate in order to improve heat conduction conditions,
- Proper heating (Figure 1b), during which the belt ends (1) are pressed against the hot plate (3) with force *F_h_*, resulting in the belt heating along its axis. This stage ends when the welding temperature *T_w_* is reached at a distance *p* from the surface of the hot plate,
- The hot plate (3) removing from the area between the belt ends (1) (Figure 1c),
- The belt connecting (Figure 1d), during which the ends of the belt (1) are pushed together at velocity *v_j_* and pressed against each other with force *F_j_*. At this stage, chemical reactions and physical interactions between the polymer macromolecules begin, resulting in the formation of a permanent connection,
- Cooling the weld (Figure 1e), during which the stiffness and strength of the weld are increased.

It should be noted that the dominant physical phenomenon associated with the belt welding process is the compression of the material under conditions of elevated temperature, whose adequate value is obtained by the heating of the belt by the hot plate. The most important physical phenomena that determine the rate of belt heating are as follows:

- Contact conduction of heat *Q_p3-1_* from the hot plate (3) to the belt (1),
- Conduction of heat *Q_p1_* inside the material volume, occurring mainly along the belt axis (1),
- Convective exchange of heat *Q_c_*_1_ between the belt (1) and the environment at temperature *T*_0_, occurring on the surface of the belt at a distance of *h* from the surface of the hot plate,
- Radiative exchange of heat *Q_r_*_1_, between the belt and the environment at temperature *T*_0_, occurring on the surface of the belt at a distance of *h* from the surface of the hot plate.

Therefore, when describing the hot plate butt welding process for belts, it is extremely important to know the thermomechanical properties of the welded material, as well as their dependence on the thermal and mechanical loads applied to the belt material.

The research is performed with regard to the welding of the belt made of thermoplastic polyurethane elastomer with trade designation TPU C85A. For this material, the welding temperature of *T_w_* is usually between 250 and 310 °C [3,4,17,18].

### 1.3. Aspects of Welding Process Research

Research works on the butt welding process of drive belts requires evaluation of the quality of the weld. Its main criterion is the results of a static test of the welded belt tension, comparing the elongation of the section of the belt with the weld to the elongation of the solid belt. The results obtained are the basis for determining the relative strength of the weld:

$$k\left(F,T_{w};v_{m};v_{j}\right)=\frac{\varepsilon_{w}}{\varepsilon_{b}}$$

where *k*—coefficient of relative strength of the weld, *F*—pressing force on the connector during the test, *T_w_*—welding temperature, *v_m_*—velocity of belt ends melting during heating, *v_j_*—velocity of joining the belt ends, *ε_w_*—relative elongation of the belt with the weld, *ε_b_*—relative elongation of the solid belt.

Research works on butt welding of plastics, taking into account the characteristics of the welded material, have been carried out multiple times. Watson [19] investigated the process of welding rigid plastics in the form of welded containers made of polypropylene. Gehde [20] conducted research on the welding of perpendicular beams made of rigid polyamide. Savija [21] studied the heat exchange in contact between the hot plate and the heated material for the purpose of welding. In turn, Stokes [22] described the process of welding rigid polyamide fittings and showed the dependence of their strength on the technological parameters of the welding process. Potente [23] investigated the welding process from the aspect of cracking of the weld after welding as a result of welding stresses. Poopat and Cocard [24,25] analyzed the optimization of welding process possibilities.

Despite the many research works that have been conducted so far, the proposed method of assessing the strength of the weld, based on deformation analysis, is not popular. Researchers typically evaluate the quality of the weld based on the maximum force or stress carried by the weld [26,27,28,29,30], additionally determining the coefficient of the relative weld strength [31]. This is due to the fact that research has usually been undertaken on the welding of relatively rigid plastics, for which the breaking load is of relatively high value and the maximum deformation during a static tensile test does not exceed a few tens of percent. Therefore, the measurement results of the force during the static tensile test are not subject to a large percentage measurement error [32,33,34,35]. In addition, the rectangular samples used in the static tensile test are less susceptible to deformation caused by the complex stress state in the joint area when compressed in the flat jaws of the universal testing machine [36,37,38].

In the case of testing of polyurethane drive belts of a circular cross-section, which have a low stiffness and strength [4,17,18], the complex stress state in the joint area of the samples results in significant deformation. In addition, such belts are susceptible to spontaneous sliding out of the grips of testing machines [39]. This phenomenon has a significant impact on the results of the study. Therefore, in this case, it is recommended to perform a static tensile test for standardized paddle samples cut out from the flat section of the material [40,41]. Unfortunately, obtaining flat samples of this shape from a belt of circular cross-section and small diameter is problematic.

Manufacturers of hot-welded polyurethane drive and conveyor belts, which are taken into account for the modeling of the hot plate welding process, offer flat belts made of the same commercial designation—TPU C85A [4,18] in addition to belts of circular cross-section. They have approximately the same selected physical properties, i.e., color and surface structure. Therefore, it is possible to make standardized samples from such a product, which could be used interchangeably for welding tests and in particular for strength tests [42,43]. However, it is necessary to investigate whether the different geometric form of the semi-finished product of such a belt, despite the declared identity of the material, has a measurable effect on its thermomechanical properties. Both types of belts were produced by continuous extrusion [5] into a semi-finished product of substantial length and then wound onto reels. 

Therefore, it is possible to hypothesize that the circular belt and the flat belt have the same thermomechanical properties, which in turn enables their interchangeable use in research work on butt welding of drive belts. 

Modeling the butt welding process requires ongoing verification of the results obtained. To this end, the impact of the plasticizing force on the deformation of the belt under conditions of increased temperature should be studied [44]. Modeling the butt welding process of drive belts requires understanding the value of the mechanical and thermomechanical properties of the belt material, in particular:

- Specific heat capacity *C_p_*,
- Thermal diffusion coefficient *a*,
- Melting temperature *T_p_*,
- Dynamic viscosity *η,*
- Density *ρ*.

Two types of samples taken from the drive belts made of the same thermoplastic polyurethane with the commercial designation TPU C85A have been analyzed [4,18]:

- Type A samples taken from the belt of circular cross-section and diameter *d* = 18 mm,
- Type B samples taken from the flat belt of the rectangular cross-section, width *b* = 140 mm and thickness *g* = 4 mm.

Therefore, the planned tests will make it possible to, first and foremost, compare the samples taken from both belt types and the verification of the research hypothesis. In addition, they will allow us to obtain quantitative data on the desired values of the thermomechanical parameters of the belt, as well as determine correction factors reducing the difference in the values for both analyzed belt types, which will make it possible to achieve the additional objective of the planned tests. 

These particular goals will be useful during the preparation of the mathematical model of the hot plate welding process. In addition, the observations made during tests will allow planning further experiments connected with this process in a better way. 

## 2. Materials and Methods

**Figure 2:**
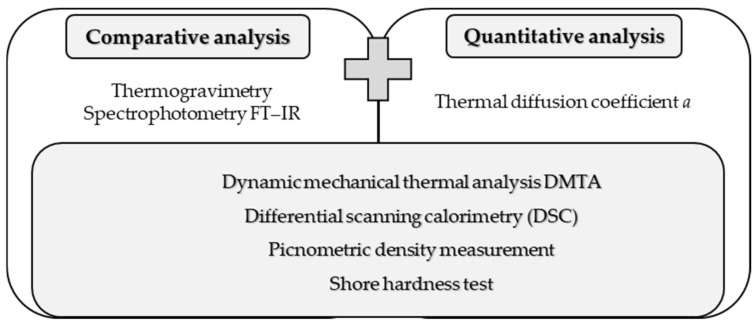
Classification of performed research.

The planned research work will enable the comparative analysis of both belt types, as well as quantitative analysis of the indicated thermomechanical parameters (Figure 2), using also dynamic parameters evaluation. Contrary to the methods known in the art for determining the mechanical properties of materials using the principles of dynamics on a macroscopic scale [45,46], the proposed research cycle will allow for an accurate comparison of samples at the structural level. The type of research was chosen in such a way as to achieve both objectives in the course of the research work. Contrary to the metrological research conducted so far in the field of drive belts, the proposed research cycle allows for comparison of both belt materials, for the purposes of e.g., designing joining technologies or conducting research works on technological processes of material processing [47].

The research work did not require chemical preparation of samples. Their geometric dimensions have been adapted to the requirements of the individual tests by:

- Machining—concurrent milling on the EMCO Concept Mill 240D numeric machine tool (EMCO GmbH, Salzburger, Austria), using cutters adapted to machining plastics,
- Plastic treatment—knife cutting using a single-shot punch.

First, comparative research was carried out to identify differences in the structure and chemical composition of samples taken from both belt types, with the aim to verify the hypothesis about the identical structure and chemical composition of the material from which both belts were made. 

The first test in this cycle is thermogravimetric analysis of samples taken from both belts. It involves introducing the test material into a state of elevated temperature, reaching a value that induces chemical decomposition. It occurs by decomposing the basic macromolecules of the polymer and the additional chemical compounds contained therein. The analysis was carried out using Netzsch TG Libra 209 (NETZSCH–Gerätebau GmbH, Selb, Germany). Testing conditions involved:

- Range of heating temperature: from +30 to +950 °C, recorded in continuous mode,
- Two types of gaseous atmosphere in which the samples were placed during the test: atmospheric air or protective gas—chemically pure nitrogen,
- The flow rate of the gas surrounding the samples was 20 cm^3^/min,
- Samples weighing 5 ± 0.2 mg,
- Heating of samples in crucibles made of aluminum trioxide.

In the next step of comparative testing, the comparative FT–IR spectrophotometric analysis was carried out for the type A sample taken from the belt of the circular cross-section and the type B sample taken from the flat belt. It entailed illuminating the belt samples with infrared radiation while simultaneously recording the radiation spectrum as it passed through the belt. The main purpose of this study was to identify the molecular structure of thermoplastic polyurethane and to compare the functional groups found in samples A and B. The analysis was carried out with the Nicolet iS50 spectrophotometer (Thermo Fischer Scientific, Waltham, MA, USA). 

Some of the most important comparative studies were the attempts at dynamic mechanical thermal analysis DMTA, which were performed in three repetitions, for both belt types, using an Anton Paar MCR302 rotational rheometer (Anton Paar GmbH, Graz, Austria). 

Two types of samples were used for testing [48,49,50,51,52]:

- Type A samples, taken from the belt of the circular cross-section and diameter *d* = 18 mm, machined into the form of rectangular beams of the following dimensions: 53.50 ± 0.31 × 10.21 ± 0.20 × 3.74 ± 0.33 mm,
- Type B samples, taken from the flat belt, guillotined into the form of rectangular beams of the following dimensions: 49.67 ± 0.13 × 9.84 ± 0.15 × 3.93 ± 0.08 mm.

Firstly, tests were conducted at room temperature. They entailed introducing controlled oscillation to rectangular samples taken from both belt types, by inflicting torsion-inducing deformations, under constant temperature conditions. Three full load cycles were performed in each repetition, assuming the following parameters:

- Test temperature of approximately 22.5 °C,
- Oscillation amplitude *A* = 0.02%,
- Oscillation frequency *f* = 1 Hz.

The measuring system of the device recorded the response of the samples to forced oscillations and current temperature conditions. On the basis of the results obtained, a comparison was made between the shear storage modulus *G*′ (lossless modulus—the real part of the shear modulus *G*) and the shear loss modulus *G*″ (lossy modulus—the imaginary part of the shear modulus *G*), the loss angle *δ*, and the dynamic viscosity *η* of both belt types.

In the second phase of DMTA tests, a series of tests were conducted under variable temperature conditions, consisting of introducing controlled oscillation to the drive belt sample, in three consecutive cycles, which were determined by alternately heating and cooling the sample. The measurement system recorded the response of the belts to forced oscillations in the form of the shear storage modulus *G*′, the shear loss modulus *G*″, the loss angle *δ*, and the value of the current temperature *T* where the sample is located. These DMTA tests were performed with the following assumptions [48,52,53]:

- Temperature range from −100 to +180 °C recorded in continuous mode,
- Rate of temperature change $\left|\nabla T\right|$ = +5 °C/min during heating and $\left|\nabla T\right|$ = −5 °C/min during cooling,
- Oscillation amplitude *A* = 0.02%,
- Oscillation frequency *f* = 1 Hz.

The purpose of this study was to compare the dynamic mechanical properties of both belts, registered under the cyclical influence of mechanical and thermal loads, and to determine their dependence on temperature values. 

The main analyzed parameter was the components of the shear modulus *G* of the belt, which results from the application of torsion loads. This methodology is typically used to define the viscoelasticity characteristics and to suppress thermoplastic materials and elastomers. In many cases, the discussed methods are used for testing susceptible structural materials or materials exposed to periodic and continuous vibration, as well as the effects of elevated or reduced temperature [54].

An important method for comparing materials, using results from DMTA, is the analysis of glass transition temperature variability *T_g_*. In this case, its value is derived from the response of the sample to cyclical mechanical and thermal loads in the area of the shear storage modulus G′, the shear loss modulus *G*″, and the loss angle *δ*. In addition, due to the specificity of the test material, which is a polyurethane elastomer, its macromolecular structure is characterized by the presence of distinguishable hard and soft segments [55,56]. Therefore, a separate analysis of glass transition temperature *T_g_^SS^* of soft and hard segments *T_g_^HS^* is also possible. These tools were used in the comparative analysis of both belt types [52,57,58].

Soft segment glass transition temperature values *T_g_^SS^* are determined on the basis of the characteristic points of the cyclical loading response [48,54,57,58,59,60]:

- Intersections of tangent relations of the shear storage modulus *G*′ and temperature,
- The maximum value of the shear loss modulus *G*″,
- The maximum value of the loss coefficient *tan δ*.

Glass transition temperature values of hard polyurethane segments *T_g_^HS^* are determined on the basis of the minimum value of the material loss angle *δ*.

The comparison of the determined values—the glass transition temperatures *T_g_^HS^* and *T_g_^SS^*, the shear storage modulus G′, the shear loss modulus *G*″, and the loss angle *δ*—allows partial verification of the hypothesis about the identical structure and chemical composition of the test belts. 

After DMTA was performed, the FT–IR spectrophotometric test was repeated for the belt samples used in the DMTA test. On this basis, a comparative analysis of the composition and chemical structure of the material, before and after the cycle of thermomechanical loads, was performed under variable temperature conditions. 

At this stage, studies that only served a comparative function were completed. The next stage of the research work consisted in the execution of tests, which was focused mainly on the recognition of the values of selected thermomechanical properties of the drive belt, which is essential in the modeling of the hot plate butt welding process of the drive belts. However, their results also provided comparative and verification information for previously completed tests.

In the next step, DSC tests were performed. The main purpose of these tests was to obtain information on phase transitions occurring during heating of samples taken from both belt types. The analysis of the results of this study was aimed at identifying the possible clear limit of material melting *T_m_* and dependence of the heat to be delivered to the material during its heating on temperature. 

The tests were divided into two stages, which were carried out using Netzch DSC 204 F1(NETZSCH–Gerätebau GmbH, Selb, Germany). Testing conditions involved the following:

- Temperature range from −80 to +300 °C recorded in continuous mode,
- Application of an inert protective atmosphere in the form of a flow of chemically pure nitrogen at a rate of 20 cm^3^/min,
- Two heating cycles at the rate of temperature change $\left|\nabla T\right|$ = +20 °C/min and one cooling cycle at the rate of temperature change $\left|\nabla T\right|$ = −10 °C/min,
- Use of samples weighing 6 ± 0.2 mg.

After the basic DSC analysis was performed, the second stage, which consists of measuring the specific heat capacity *C_p_*, was done. Information about the value of this parameter is important from the point of view of modeling the temperature distribution during heating of the drive belt in the technological process of butt welding. In this case, testing conditions involved the following:

- Temperature range from 0 to 150 °C, with a narrower test temperature range compared to the basic DSC analysis, due to the need of ensuring the absence of phase transitions of the material that would impair the correct determination of the specific heat *C_p_*, which is also due to apparatus requirements,
- Application of a protective inert atmosphere in the form of chemically pure nitrogen with a flow rate of 20 cm^3^/min,
- One heat cycle at the rate of temperature change $\left|\nabla T\right|$ = +20 °C/min,
- Use of the reference material in the form of synthetic sapphire (α-A_2_O_3_) with the mass similar to that of the analyzed samples.

The specific heat capacity *C_p_* of the samples taken from the circular cross-section belt (A) and the flat belt (B) was taken in a normalized way [61], based on the recorded change in the enthalpy of the polymer during heating. 

**Figure 3:**
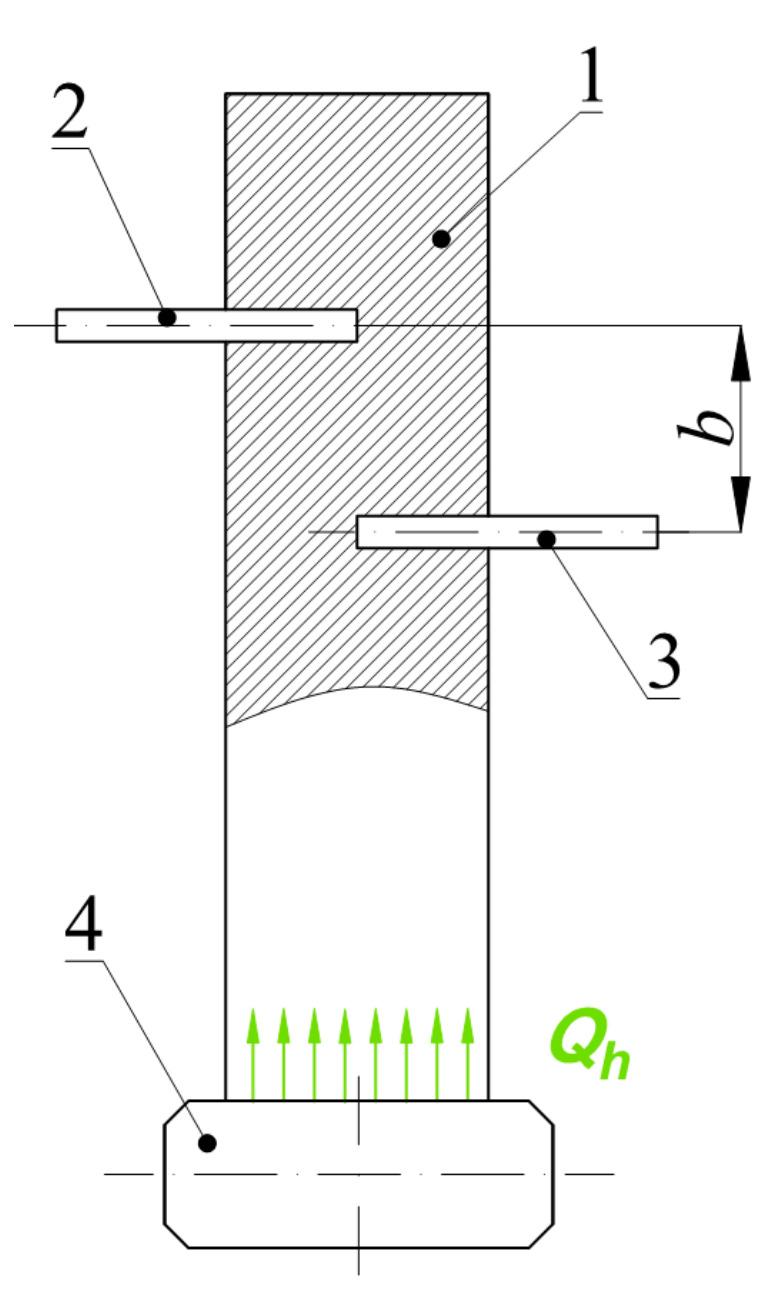
Method of measurement of thermal diffusion coefficient: 1—sample, 2 and 3—thermoelectric cells, 4—heating plate; *b*—known distance between thermoelectric cells, *Q_h_*—heat exchanged by conduction between plate and belt.

Next, the thermal diffusion coefficient *a* was determined (Figure 3). The test consisted of heating the belt sample in the form of a rectangular beam (1) by applying the heater (4) to one of its flat surfaces and simultaneously measuring the temperature values at two measurement points, at a known distance *b* between them. The temperature was measured using thermoelectric cells (2 and 3), which were inserted into the holes made in the test belt sample. 

The assumptions for the study of thermal diffusion *a* were as follows:

- Isotropic nature of heat flow inside the sample,
- Heating the sample in a low-pressure atmosphere to reduce the effect of convection on sample heating,
- Thermoelectric cells were introduced to half the depth of the sample,
- Maximum heater temperature *T_g_* = 100 °C,
- Contact surfaces between the heater and the sample, as well as thermoelectric cells, and the sample connected by a thermal conductive paste with the heat conductivity coefficient *λ =* 0.88 W/m∙K,
- Maximum heating time *t_g_* = 300 s,
- The distance between the thermoelectric cells *b* = 5 ± 0.1 mm and was measured for each sample tested.

In the case of thermal diffusion coefficient analysis, only the sample taken from the flat belt was subjected to the test consisting of five repetitions due to apparatus conditions. Therefore, the experiment was not of a comparative nature and only served to quantify this thermomechanical parameter.

At the end of the test cycle, 2 types of tests were performed, which were oriented toward the determination of the selected mechanical parameters of the two studied belt samples:

- The density of the material according to the picnometric method, in a normalized manner [62]. The material was shredded into sections of the following size: 10 mm for sample A and 5 × 30 mm for sample B. A 25 cm^3^ glass picnometer was used for determination. The environment temperature at the time of determination was *T*_0_ = 22.5 °C,
- Standard Shore hardness test, using the Bareiss hardness meter (HP, Bareiss Prüfgerätebau GmbH, Oberdischingen, Germany), with a maximum value indicator [63].

The results of the test cycle carried out in this way provided information on the values of the selected thermal properties of the belt material for both sample types and were used to verify the hypothesis of the possibility of interchangeable use of samples taken from the belt of circular cross-section (A) and the flat belt (B) for the research work on the hot plate butt welding of drive belts. 

## 3. Results

**Figure 4:**
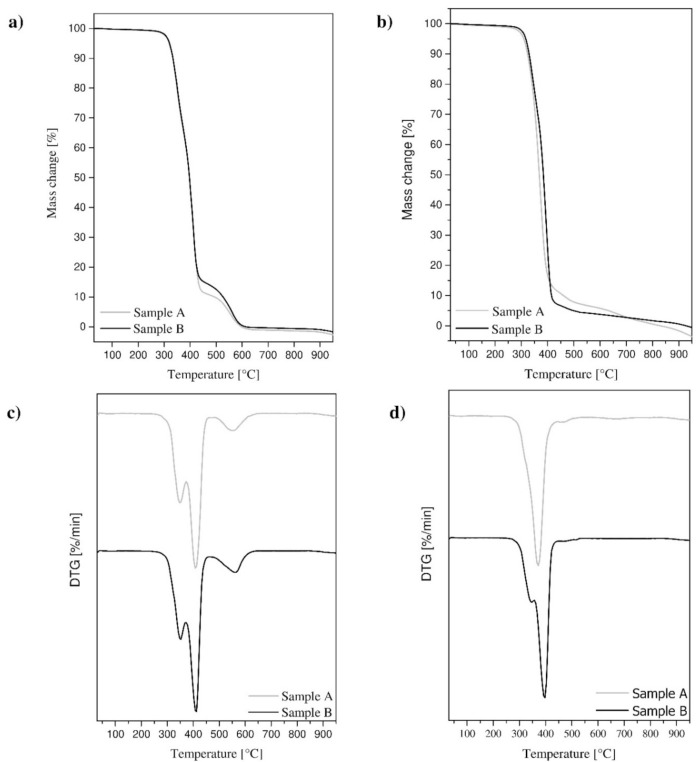
Thermogravimetric curves made in the atmosphere of a flowing gas: air (**a**) and nitrogen (**b**) and derivatographic curves in the atmosphere of a flowing gas: air (**c**) and nitrogen (**d**).

Thermogravimetric analysis performed in the atmospheric air setting has shown that the mass loss of sample B totaling 1% occurs at temperature *T_mr_*_1%_ with a higher value than for sample A (Table 1, Figure 4a). 

The process of decomposition of the material, surrounded by atmospheric air, took place in three stages for both analyzed samples (Figure 4c). The beginning of the first and second stages of polymer structure decomposition, for sample B taken from the flat belt, occurs at a higher temperature *T_vmr_* than for sample A. However, it should be noted that the difference between these values is relatively small and does not exceed 1%. The third decomposition stage, for both samples, has a lower energy level. Temperature *T_vmr_* of the maximum mass change rate for sample B for all three decomposition steps, compared to sample A, is about 1–2% higher, with the largest difference observed for the last decomposition step of the material. Thermal analysis performed in the protective atmosphere of chemically pure nitrogen also showed greater chemical stability of sample B (Figure 4b,d). However, in this case, the temperature difference *T_mr_*_1%_ at 1% mass loss for the two analyzed samples is greater and is approximately 25%. The decomposition process for sample A is one step, whereas for sample B, it is two steps. Temperature *T_vmr_* at the maximum rate of change of mass for sample B is also higher than that for sample A. In this case, the difference is about 7%, although for sample A, this phenomenon occurs in the first and only decomposition step. 

**Figure 5:**
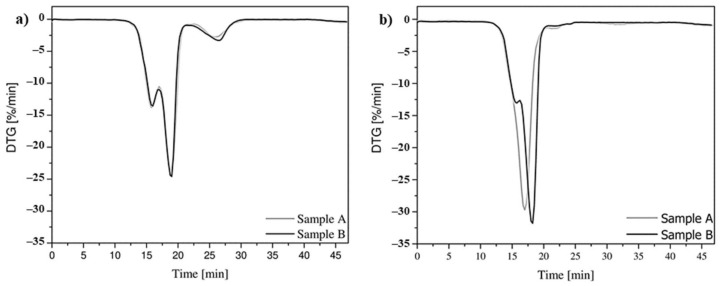
Derivatographic curves made in the atmosphere of a flowing gas: air (**a**) and nitrogen (**b**).

Thermogravimetric analysis also allowed the determination of the temperature *T_vmr_* at the maximum rate of change in mass, depending on time *t_vmr_* (Figure 5, Table 1). In the atmosphere of flowing air, both analyzed samples decompose in similar time *t_vmr_*. However, in a protective inert atmosphere, the decomposition time *t_vmr_* for both samples varies. However, it should be noted that it is proportional to temperature *T_vmr_* at which the next step of decomposition of the polymer occurs. 

**Figure 6:**
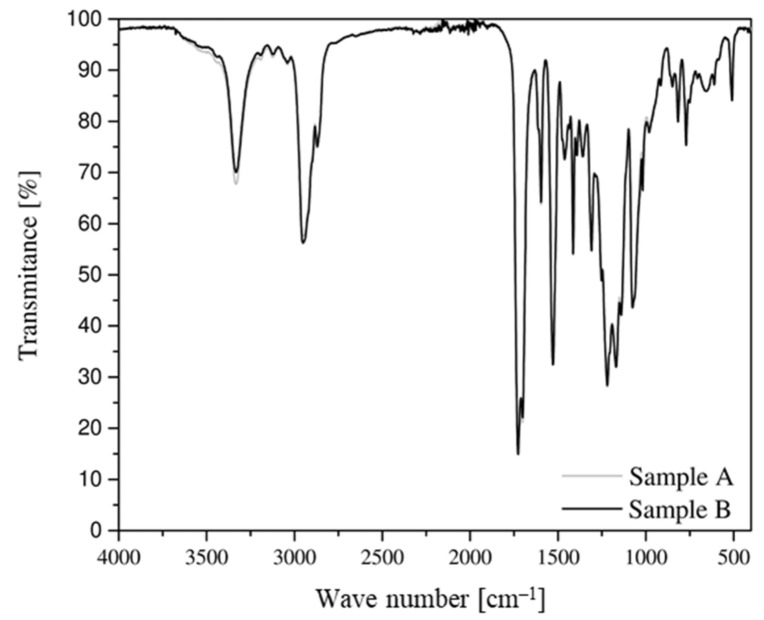
Absorption spectrum for the material of drive belt samples taken from both belt types, which was performed during a spectrophotometric test.

The results of the FT–IR spectrophotometric analysis for both belt types are very similar, as evidenced by the almost overlapping radiation spectra (Figure 6). For both samples, radiation spectra from N-H stretching vibrations were observed. At a wave number of 3325 cm^−1^, the effects from the vibration of the N-H group associated with H are present, while the light effect at 3450 cm^−1^ (for free N-H groups) is negligible, indicating that almost all N-H groups are bound. In the wave number range of 3000–2800 cm^−1^, there are effects denoting stretching C-H aliphatic hydrocarbons. At a wave number of 1750–1600 cm^−1^, spectra derived from the tensile interactions of the CO group are observed [52].

DMTA conducted at room temperature showed a difference between samples A and B in the shear storage modulus *G*′. The sample taken from the circular cross-section belt (A) has values higher by about 50% in the first load cycle and 70% in the second and third load cycles (Table 2). 

For both types of samples, the first load cycle is followed by a decrease in the value of the shear storage modulus *G*′. The next two cycles have similar values of this parameter. When analyzing the results for the sample taken from the belt of circular cross-section (A), the value of the shear storage modulus *G*′ decreases by approximately 18% after the first load cycle, representing a clear difference. In contrast, the difference between the second and third load cycles is less than 5%. 

In the case of samples taken from the flat belt (B), the conduct of the shear storage modulus *G*′ over successive load cycles has a similar dependence to that of the circular cross-section belt (A). 

For both types of samples tested, the variability of the shear loss modulus *G*″ and the dynamic viscosity *η*, depending on the current load cycle and sample type, is almost identical to that of the shear storage modulus *G*′. The only difference is the slightly different course of the loss angle *δ*.

**Figure 7:**
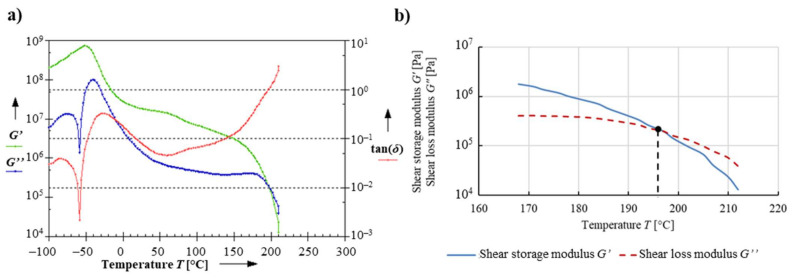
Determination of the temperature range for the DMTA test, (**a**) the result of the analysis in the temperature range from −100 to 210 °C, (**b**) determination of the intersection point of the shear storage modulus *G*′ and the shear loss modulus *G*″.

The temperature range of DMTA for variable temperature conditions was determined after the first trial test tor sample B, which was conducted in the range from −100 to 210 °C (Figure 7a). It follows that the test material undergoes a phase transition to a plasticized phase at 197 °C (Figure 7b), when the shear loss modulus *G*″ takes the value greater than the shear storage modulus *G*′. At this time, the loss angle *δ* exceeds 45°, and the phenomenon of plastic flow begins in the material.

**Figure 8:**
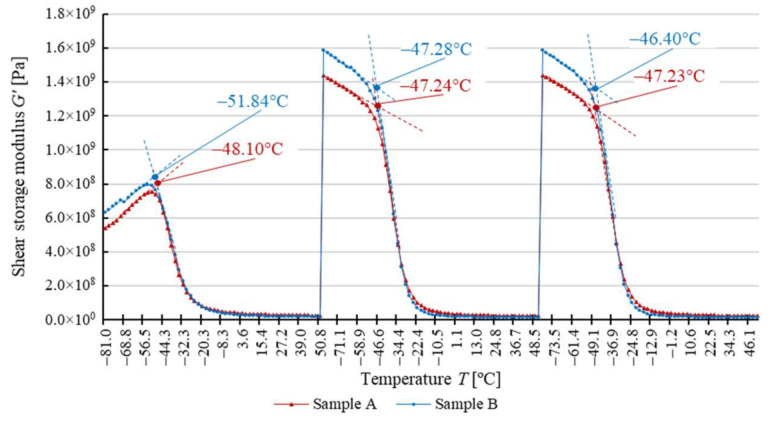
Determination of polyurethane glass transition temperature based on the shear storage modulus *G*′.

**Figure 9:**
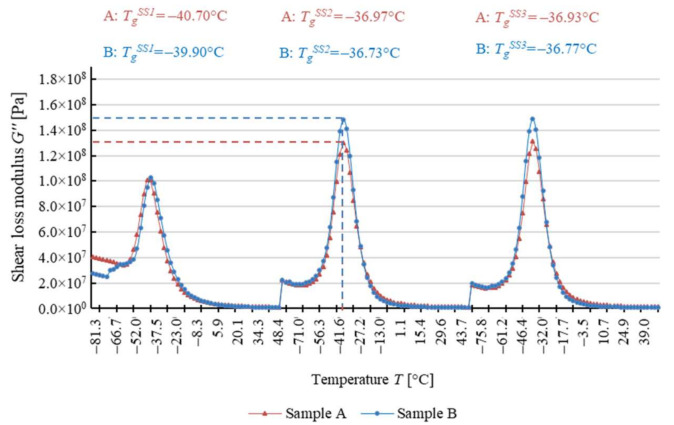
Determination of polyurethane glass transition temperature based on the shear loss modulus *G*″.

**Figure 10:**
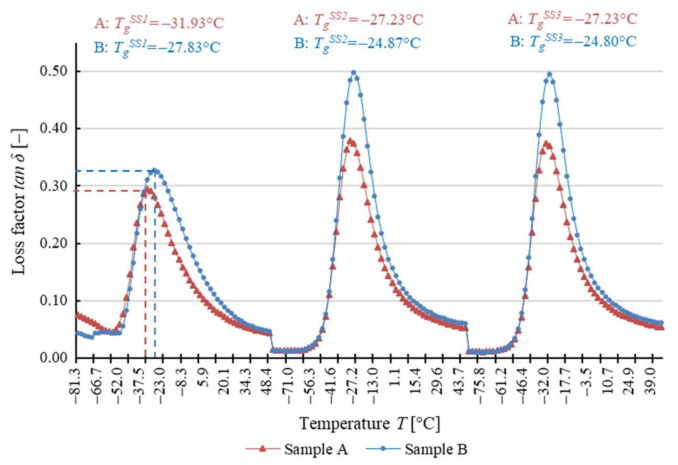
Determination of polyurethane glass transition temperature based on the loss factor tan *δ*.

The dependence of the response to cyclical torsion loads on temperature for both types of test samples is shown in Figure 8 due to the shear storage modulus *G*′, in Figure 9 due to the shear loss modulus *G*″, and in Figure 10 due to the loss angle *δ*. The two types of samples exhibit highly similar results, as evidenced by the analyzed dependence of these parameters on temperature values. 

The glass transition temperature of soft segments of analyzed polyurethane *T_g_^SS^* ranges from about −25 °C to about −50 °C (Table 3). After the first heating and cooling cycle, the temperature *T_g_^SS^* determined in relation to the shear storage modulus *G*′ is slightly higher for sample A than for sample B (Figure 8), with the difference not exceeding 8%. The reverse phenomenon was observed with *T_g_^SS^* determined relative to the shear loss modulus *G*″ (Figure 9), with the value lower by about 2% for sample A. For the loss factor *tan δ* (Figure 10), the glass transition temperature *T_g_^SS^* for sample A is approximately 10% lower. At the same time, it should be noted that this is the maximum difference between the determined glass transition temperature *T_g_^SS^* values throughout the test. 

It is important that in the determined glass transition temperature *T_g_^SS^* values, there is a phenomenon analogous to that of the constituent of shear modulus *G*′ and *G***″** and the loss angle *δ* (Table 3). It entails changing the values of each of these parameters after the first heating and cooling cycle, which is combined with the cyclical oscillations of the sample. After the first test cycle, the glass transition temperature *T_g_^SS^* for both samples increases. The shear storage modulus *G*′ and the shear loss modulus *G***″** as well as the loss angle *δ* change in a similar way. However, after the second test cycle, the values remain approximately constant. 

The glass transition temperature of hard polyurethane segments *T_g_^HS^*, determined by the minimum value of the loss angle *δ*, is characterized by an increase in value between the first and the second heating and cooling cycle, as is the glass transition temperature of soft segments *T_g_^SS^*. Similar to previous DMTA tests, the glass transition temperature *T_g_^HS^* during the first cycle is higher for the circular cross-section belt (sample A) than for the flat belt (sample B) by about 10%, with the difference significantly decreasing with the subsequent heating and cooling cycles of the sample (Table 4).

**Figure 11:**
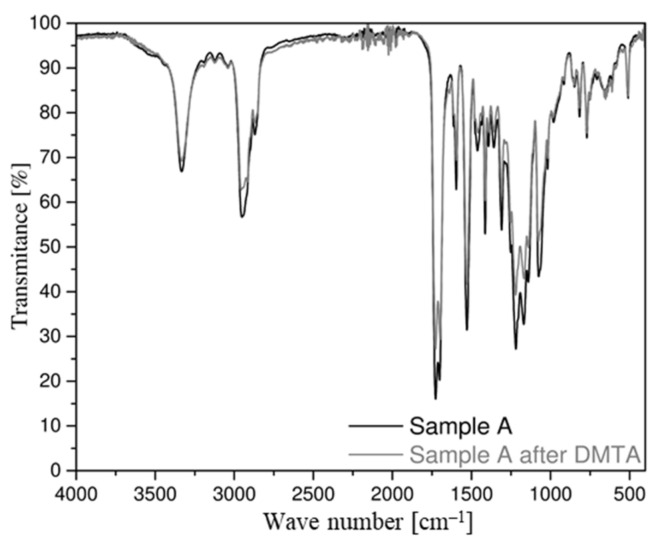
Comparison of the absorption spectrum for the sample A of the drive belt.

**Figure 12:**
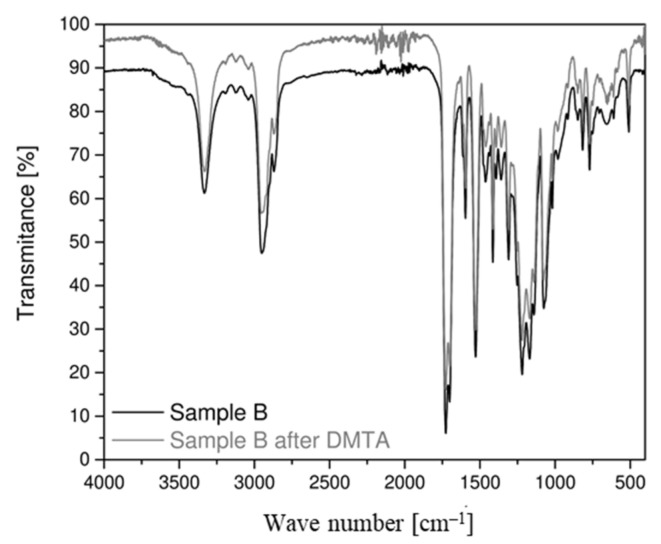
Comparison of the absorption spectrum for the sample B of the drive belt.

After DMTA was performed, FT–IR spectrophotometric testing was performed again. On this basis, comparative spectra were produced for belt samples before and after they were subjected to thermomechanical load cycles. When comparing the FT–IR spectra of samples A and B before and after the DMTA test, no significant differences in the shape of the spectrum is noticeable (Figure 11 and Figure 12). Only a slight change in transparency is noticeable, especially in the case of the sample taken from the flat belt (B).

**Figure 13:**
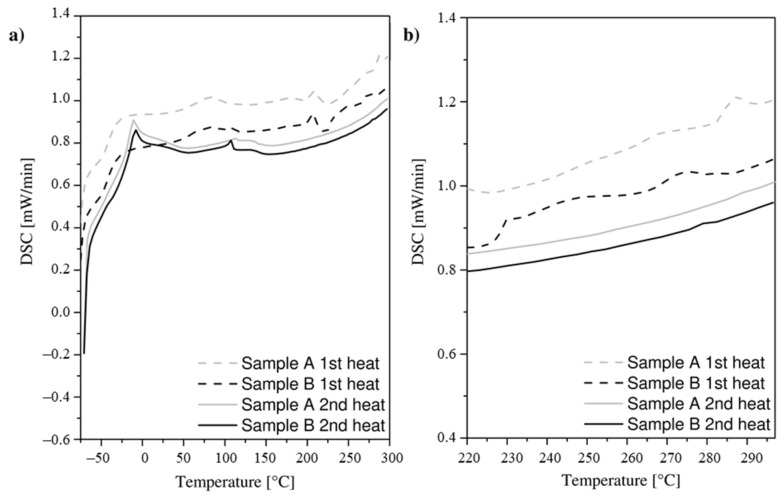
DSC curves in the full range of test temperature values (**a**) and approximately per range corresponding to the welding temperature values *T_w_* (**b**).

DSC analysis carried out in the atmosphere of the inert gas showed the presence of a distinct endothermic effect of both materials at temperatures below 0 °C, indicating the presence of a vitrification (Figure 13, Table 5).

Analyzing the course of the first heating of sample A, the following endothermic effects were observed:

- Glass transition temperature of soft polyurethane segments *T_gA_*_1_*^SS^* = −38.1 °C,
- Glass transition temperature of polyurethane hard segments *T_gA_*_1_*^HS^* = 63.2 °C,
- Additional endothermic effect *T_mpA_*_1_ = 211.5 °C.

Analyzing the course of the second heating of sample A, the following endothermic effects were observed:

- Glass transition temperature of soft polyurethane segments *T_gA_*_2_*^SS^* = −30.2 °C,
- Glass transition temperature of polyurethane hard segments *T_gA_*_2_*^HS^* = 112.2 °C,
- No additional endothermic effect at a higher temperature.

Analyzing the course of the first heating of sample B, the following endothermic effects were observed:

- Glass transition temperature of soft polyurethane segments *T_gB_*_1_*^SS^* = −37.4 °C,
- Glass transition temperature of polyurethane hard segments *T_gB_*_1_*^HS^* = 72.1 °C,
- Additional endothermic effect *T_mpB_*_1_ = 208.3 °C.

Analyzing the course of the second heating of sample B, the following endothermic effects were observed:

- Glass transition temperature of soft polyurethane segments *T_gB_*_2_*^SS^* = −33.9 °C,
- Glass transition temperature of polyurethane hard segments *T_gB_*_2_*^HS^* = 108.1 °C,
- No additional endothermic effect at a higher temperature.

It should be noted that the course of the first response of the sample to thermal loads during the heating of both samples is slightly different than in the subsequent cycle. On the recorded runs of the DSC curve during the second heating, smoothing of the supplied heat energy as a function of temperature was observed for both samples tested. In addition, during the second heating, the endothermic effect occurring above 200 °C disappears.

In the case of higher temperature values, exceeding 220 °C, at which the plasticity of the material normally occurs in the butt welding process of the drive belts (Figure 13b), no significant differences were observed between comparable belt samples. No endothermic effect was observed in this range. 

**Figure 14:**
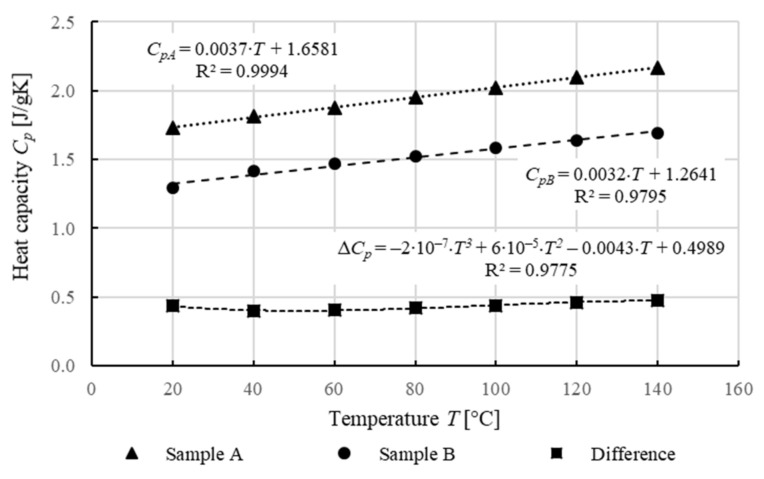
Dependence of specific heat capacity *C_p_* [J/gK] on temperature.

During the DSC test, the specific heat capacity *C_p_* was also determined for the two studied belt samples (Figure 14 and Table 6). 

Sample A exhibits higher specific heat capacity *C_p_* over the entire temperature range compared to sample B. Based on the analysis of the change in the specific heat capacity of the samples tested as a function of temperature values, the following can be inferred:

- The specific heat capacity *C_p_* in both cases increases in a linear manner as the temperature increases,
- The difference in the specific heat capacity *C_p_* for both samples is almost constant throughout the test temperature range.

The thermal diffusion coefficient of the material is *a* = 5.76∙10^−7^ ± 1.29∙10^−7^ [m^2^/s]. 

The material density for sample A is 1.1901 ± 0.0008 g/cm^3^; whereas for sample B, it is 1.1914 ± 0.0009 g/cm^3^. Therefore, the difference between the two materials is relatively small. 

The Shore hardness for both materials has a similar value, respectively for sample A = 90.45 ± 0.52°ShA, for sample B = 90.36 ± 0.67°ShA. 

## 4. Discussion

Thermogravimetric analysis performed in the atmospheric air setting showed greater thermal stability of sample B. Therefore, when the material is heated, the first phase of material decomposition occurs slightly later. The decomposition of the material in the case of both samples occurs in three stages, the first involving the decomposition of soft segments of thermoplastic polyurethane, the second involving the decomposition of hard segments, and the third involving the degradation of inorganic residues in the material. The differences in the decomposition temperature of the two samples tested are relatively small and not noticeable during industrial welding of drive belts. It should also be noted that the decomposition of the material occurs at temperatures well above the welding temperature *T_w_*. This is justified because during welding, the chemical decomposition of the material cannot occur [64,65].

Thermogravimetric analysis in the inert atmosphere also demonstrated greater chemical stability of sample B. However, in this case, the decomposition process for sample A is one step, whereas for sample B it is two steps, which is a significant difference from the decomposition in the atmospheric air setting. It should also be noted that the temperature values at which the different key stages of decomposition of the two polymers take place are lower than in the atmospheric air setting. Therefore, in an inert atmosphere, the material under study exhibits different behaviors during heating than in the atmospheric air environment. 

According to the thermogravimetric analysis, the differences between the materials analyzed, in the expected temperature range of *T_w_* between 250 and 310 °C are small enough to be negligible. Significant discrepancies between these materials occur at higher temperatures, near the first stage of material decomposition, and at higher temperatures. However, it should be noted that from the point of view of the belt butt welding process, these discrepancies are irrelevant. 

The gas surrounding the material also has a significant effect on its behavior during heating. In an inert atmosphere, some structural differences between the two belt types are highlighted, and this should therefore be taken into account in the design of welding technology in a protective atmosphere of this type. Such work has already been carried out, and it has been proved that this technology requires a special approach [66]. Similarly, forcing the flow of air cooling the weld changes the heat transfer conditions during welding and requires taking this phenomenon into account when designing this technology [67]. It should be noted that in the case of the classical butt welding process of drive belts, conducted in the atmospheric air setting, the response of the material to the forced temperature change is more predictable and repetitive, and the resulting change in the material properties for both belt types is exhibiting a smaller difference.

In view of the performed analysis, based on the results of the thermogravimetric test, from the point of view of the butt welding process of the drive belts at temperature *T_w_*, without the application of an additional protective atmosphere, it is appropriate to state that both studied belts have the same characteristics, and it is not required to make a distinction between them. 

FT–IR spectrophotometric analysis revealed that the absorption spectra for sample types are nearly identical. Therefore, the composition and chemical structure for both materials are the same. 

Summarizing the results obtained by thermogravimetric and spectrophotometric analysis, it should be noted that both materials are identical in terms of chemical composition and molecular structure. 

Comparative DMTA tests, performed at a constant standard temperature of material usage, indicate the difference between the dynamic properties of both studied drive belt types. The component values of the shear modulus *G* and dynamic viscosity *η* indicate higher stiffness of the circular cross-section belt, which is due to the greater ordering and axial orientation of the polymer chains during extrusion, due to the smaller geometric dimensions of the product [68,69,70]. 

The decrease in the value of the shear modulus *G* and dynamic viscosity *η* for both belts is due to the material structure relaxation processes that occur after the first cycle. Under the influence of cyclically applied mechanical stresses, the chains are ordered, and the entropy of the system is reduced. As a result, the material obtains a stable structure, which is characterized by the fact that the values of the mechanical properties do not change with the passage of successive load cycles [52,69,70,71].

As with most polymer materials, at temperatures lower than the glass transition temperature *T_g_*, the material exhibits the capability for brittle cracking, while above *T_g_*, the material undergoes significant deformation, with decreasing force causing deformation. When the glass transition temperature is exceeded, there is a fundamental change in the macroscopic properties of the polymer, from the vitreous solid state to a highly elastic material, with a further increase in temperature resulting in a plastic flow effect [56,57]. 

In the case of DMTA for variable temperature, materials have shown comparable glass transition temperature values for soft segments *T_g_^SS^*, and the relatively small differences observed during the first heating cycle disappear in subsequent cycles. Given that such a result has been obtained for the three methods of determination, it should be assumed that the glass transition temperature is approximately the same for both studied belts. This indicates that these materials have a similar response to dynamic loads under changing temperature conditions, which proves that their microstructure and chemical composition are identical. This phenomenon occurs regardless of the number of cycles of thermal and mechanical loads to which the belt has been subjected.

The observed increase in the shear storage modulus *G*′ and the shear loss modulus *G*″, as well as the loss angle *δ* and the associated glass transition temperature of soft segments *T_g_^SS^*, occurring to a comparable extent for both belt types, as a function of successive cycles of thermal load change, follows from two findings:

- The migration of plasticizing additive or additional chemical compounds added to polyurethane (diisocyanates, polyols, chain extenders) that takes place after the sample is heated in the first cycle [72,73],
- The processes of material structure relaxation that take place after the first cycle. As in the case of tests at the standard temperature of material usage [68], as a result of mechanical and thermal stresses cyclically applied, the sequencing of the chains and reduction of the entropy of the system occurs [52,55,71,73].

It should be noted that the change in the measured mechanical parameters and the glass transition temperature *T_g_^SS^* occurs to the same extent for all the methods of analysis presented (analysis of the shear storage modulus *G*′, the shear loss modulus *G*″, and the loss angle *δ*), which indicates the correct selection of the test methodology [48,54,57,58,59,60]. 

In the case of analyzing the glass transition temperature of hard segments *T_g_^HS^*, as in the case of soft segments, an increase after the first load cycle and stabilization thereafter was observed in both cases. The reasons for this phenomenon are the same as for soft segments [54]. The initial difference in the glass transition temperature of the hard segments *T_g_^HS^* of the material of both belts is indicative of the initial higher stiffness of the circular cross-section belt. However, this difference is small and disappears over successive cycles of thermal and mechanical loading. 

Therefore, in terms of mechanical properties, under dynamic load conditions and variable temperature, these belts are identical. This assumption is particularly correct because of the specificity of the butt welding process of drive belts, in which heat is supplied to the belt material, resulting in a higher welding temperature *T_w_* than the temperature in DMTA tests. Therefore, the migration processes of the plasticizing additive and stress relaxation occurring during welding will cause the discrepancy between both belt samples to be imperceptible. 

According to the FT–IR comparative spectrophotometric analysis, both for the flat belt (B) and the circular belt (A) samples, there is no change in the composition or structure of the chemical bonds after cyclically loading the samples under variable temperature conditions. Therefore, it is expected that after butt welding, the material will retain most of the properties resulting from its structure and chemical composition.

The thermal degradation of thermoplastic polyurethane is a process in which soft and hard segments may behave differently during heating. Analysis by DSC carried out in an inert gas atmosphere revealed the presence of glass transition phenomenon for both samples analyzed both for soft segments *T_g_^SS^* and hard segments *T_g_^HS^* [74,75,76]. 

During the first heating cycle, for both sample types, in addition to unambiguous glass transition temperature values of soft segments (*T_gA_*_1_*^SS^* and *T_gB_*_1_*^SS^*) and hard segments (*T_gA_*_1_*^HS^* and *T_gB_*_1_*^HS^*), an endothermic effect was observed at temperatures *T_mpA_*_1_ and *T_mpB_*_1_. It is caused by the decomposition and migration of the plasticizing additive contained in the structure of the test samples. This phenomenon is irreversible because it is not observed during the second heating cycle. It causes the glass transition temperature (*T_gA_*_2_*^SS^*, *T_gB_*_2_*^SS^*, *T_gA_*_2_*^HS^* and *T_gB_*_2_*^HS^*) to increase during the second heating cycle. The presence of the plasticizing additive is justified because it improves the susceptibility to plastic processing of the polymer by lowering the second-order transition temperature (*T_g_*) and is added to the material for the manufacturing process of continuous extrusion. However, it should be noted that the increased temperature causes its effect to fade and thus does not alter the properties of the target product [72,73]. 

During the second heating cycle, for both sample types, only thermal effects occurring at the glass transition temperature of soft segments (*T_gA_*_2_*^SS^* and *T_gB_*_2_*^SS^*) and hard segments (*T_gA_*_2_*^HS^* and *T_gB_*_2_*^HS^*) were observed. The lower intensity of the endothermic effect from the glass transition of soft segments in sample B taken from the flat belt is due to the lower concentration of this phase [77,78,79].

The nearly identical results of the DSC tests for both belt types and for temperatures exceeding 220 °C show the same effect of increased temperature on the behavior of both belt types in this range. In addition, it should be noted that in the case of both samples, no endothermic effect can be observed, indicating the presence of a clear melting limit of the material expected at a temperature exceeding 220 °C. This shows that this material, as a result of the networking and characterization of the macromolecular structure, behaves in a similar way to an amorphous body—in which there is no clear melting limit [55,56,80,81]. As a result, it is impossible to accurately determine the melting point, the value of which must be exceeded, to begin the butt welding process of the drive belts, as confirmed in [43], among other publications. 

The difference in the specific heat capacity *C_p_* for both studied samples, as for the components of the shear modulus *G* and dynamic viscosity *η* determined during DMTA at a constant temperature, is due to the different degree of ordering of the polymer chains in both belt types, which is closely related to their method of manufacturing and geometric form. Drawing a belt with a circular cross-section and relatively smaller dimensions makes the polymer chains more ordered than in the case of the flat belt. Therefore, the belt material of circular cross-section, when formed, reaches a lower energy level [68,69,70,81]. Thus, to heat it up to a certain temperature and bring it to a different energy level, more energy is required, resulting in more heat to be delivered to the unit of mass. The difference of the specific heat capacity *C_p_* must be taken into account when modeling the heat flow of the welding process. When developing a mathematical model, by changing the type of belt being analyzed, an appropriate correction factor for the specific heat coefficient *C_p_* should be assumed.

Determination of the glass transition temperature of the tested polymers *T_g_* using DMTA indicates values higher than in DSC, which is due to the specificity of the apparatus. A comparison of the results obtained from DSC and DMTA confirms differences in the measured glass transition temperatures of about 10 °C. In addition, it should be noted that both tests (DMTA and DSC) confirm the presence of the same phenomena occurring under the influence of thermal and mechanical loads [82,83]. Thermal effects on the belt material cause irreversible changes by the migration or decomposition of additives, resulting in improved plastic properties. In turn, cyclical mechanical loads initiate the phenomenon of stress relaxation [52]. These phenomena affect the change in the thermomechanical properties of the material of both studied belt types occurring after the first cycle of thermal or mechanical load. During further application of mechanical or thermal loads, in both cases, the material does not change its properties—the values of its selected thermomechanical parameters are stabilized. In addition, in this case, the differences between both analyzed belts are blurred. 

The measured hardness and density values are typical for this type of polyurethane material. This type of polyurethane has a hardness higher by about 10°ShA than standard rubber materials. Both the flat belt and the circular cross-section belt have almost identical values [52,84,85].

## 5. Conclusions

Comparative analysis of the TPU samples, which were taken from the belt with a circular cross-section (A) and from the flat belt (B), conducted using a set of thermomechanical parameters measurement, provides the following observations:

- At the maximum temperature *T_w_*, which reaches 310 °C, during the hot plate welding carried out in the atmospheric air, the structural changes in both belts materials, from the thermal decomposition point of view, are negligible. Therefore, the thermal decomposition phenomenon can be omitted during the analysis of the welding process, because the temperature values in which changes are noticeable are not reached,
- Both of the belts have the identical structure and chemical composition,
- Considering cyclic loads for both types of the belt, the shear modulus *G* and the dynamic viscosity *η* at room temperature change their values, as a result of stress relaxation after the first applied load, and they remain constant during subsequent cycles of loading. Adding the temperature variation causes these parameters, for both types of the belt, to become uniformed, as a result of stress relaxation and migration of the plasticizing additives. Therefore, as regards the hot plate welding process, which involves the compression of the belt under elevated temperature conditions, the difference between a circular (A) and a flat belt is negligible (B),
- As a result of cyclical mechanical and thermal loads, the basic chemical structure of the material of both belts does not change,
- Once heated to the same degree, both belts harmonize their properties with respect to mechanical response for cyclical loads and the energy demand of the heating process. These factors cause the properties of the belt during the welding process becomes more predictable,
- Both: circular (A) and flat (B) belts do not show a clear melting limit,
- Both samples differ in the specific heat capacity *C_p_*; however, this difference is constant, and thanks to that, it can be taken into account when modeling the welding process in an easy way,
- Both the circular (A) and flat (B) cross-section belts have approximately equal density and hardness.

On the basis of these conclusions, the hypothesis about the possibility of interchangeable use of samples of both belts for hot plate butt welding research can be confirmed, assuming the following:

- Welding temperature *T_w_* higher than 220 °C and lower than 310 °C,
- Welding without the use of inert protective atmospheres,
- The application of the correction factor ∆*C_p_*, for specific heat capacity *C_p_*, to model the heat flow of the process.

It is also extremely important that the change in the temperature of the material with the simultaneous application of mechanical loads does not result in chemical and structural changes in the material. Therefore, it can be concluded that on a macroscopic scale, the process of welding of this material does not result in the formation of the weld with significantly different properties relative to the solid belt for both drive types.

It is extremely important that the proposed set of thermomechanical tests can be used to perform comparative tests of various products made of thermoplastic elastomers, the purpose of which is to analyze the possibility of an interchangeable use of samples in research.

## Figures and Tables

**Table 1:** Thermogravimetric analysis results.

Atmosphere	Sample	Temperature at 1% Mass Loss *T_mr_*_1%_ [°C]	Temperature at Maximum Mass Change Rate *T_vmr_* [°C]			Time at Maximum Mass Change Rate *t_vmr_* [min]		
			1. Stage	2. Stage	3. Stage	1. Stage	2. Stage	3. Stage
Atmospheric air	A	270.1	349.2	409.5	549.8	17.63	20.58	27.58
	B	275.1	350.1	410.7	563.5	17.76	20.67	28.26
Nitrogen (N_2_)	A	215.6	371.2	–	–	18.70	–	–
	B	270.2	346.6	395.8	–	17.59	20.02	–

**Table 2:** Values of: shear storage modulus *G*′, shear loss modulus *G*″, dynamic viscosity *η* and loss factor *tan δ* determined at room temperature.

Sample	Cycle	Temp *T* [°C]	*G*′ [MPa]	*G*″ [MPa]	*η* [MPa⸳s]	*tan δ* [–]
A	1	22.47 ± 0.02	29.40 ± 1.55	2.03 ± 0.23	4.69 ± 0.25	0.0669 ± 0.0041
	2	22.43 ± 0.02	24.87 ± 3.75	1.90 ± 0.31	3.97 ± 0.60	0.0763 ± 0.0009
	3	22.43 ± 0.02	26.00 ± 3.59	2.02 ± 0.31	4.15 ± 0.58	0.0776 ± 0.0011
B	1	22.47 ± 0.02	18.90 ± 0.62	1.58 ± 0.056	3.02 ± 0.10	0.0840 ± 0.0004
	2	22.43 ± 0.02	13.60 ± 0.89	1.17 ± 0.093	2.17 ± 0.14	0.0862 ± 0.0014
	3	22.47 ± 0.02	13.90 ± 0.95	1.22 ± 0.095	2.22 ± 0.15	0.0874 ± 0.0016

**Table 3:** *T_g_^SS^* values determined in DMTA test.

Sample Type	Cycle	*T_g_^SS^* [°C] by *G*′	Module *G*′ [MPa] at *T_g_^SS^*	*T_g_^SS^* [°C] by *G*″	Max *G*″ [MPa]	*T_g_^SS^* [°C] by *tan δ*	Max *tan δ* [–]
A	1	−48.10 ± 4.31	848.80 ± 115.68	−40.70 ± 2.77	108.33 ± 1.53	−31.93 ± 2.30	0.2963 ± 0.0057
	2	−47.24 ± 0.56	1233.28 ± 114.83	−36.97 ± 0.25	130.33 ± 10.41	−27.23 ± 0.12	0.3783 ± 0.0090
	3	−47.23 ± 0.56	1248.15 ± 110.62	−36.93 ± 0.21	131.33 ± 10.21	−27.23 ± 0.12	0.3740 ± 0.0079
B	1	−51.84 ± 3.84	861.81 ± 146.01	−39.90 ± 2.77	103.87 ± 12.71	−27.83 ± 1.10	0.3273 ± 0.0051
	2	−47.28 ± 0.20	1369.58 ± 29.11	−36.73 ± 0.13	148.67 ± 3.51	−24.87 ± 0.06	0.4987 ± 0.0064
	3	−46.40 ± 0.04	1374.80 ± 33.89	−36.77 ± 0.06	149.00 ± 3.00	−24.80 ± 0.01	0.4943 ± 0.0067

**Table 4:** *T_g_^HS^* values determined in DMTA test.

Sample	Cycle	*T_g_^HS^* °C by *tan δ*	Min *tan δ*
A	1	65.37 ± 5.84	0.0425 ± 0.0013
	2	74.67 ± 3.55	0.0476 ± 0.0004
	3	74.70 ± 2.69	0.0487 ± 0.0003
B	1	57.76 ± 0.06	0.0452 ± 0.0012
	2	73.03 ± 3.61	0.0555 ± 0.0004
	3	70.03 ± 1.27	0.0571 ± 0.0004

**Table 5:** Glass transition temperature *T_g_* and additive endothermic effect temperature *T_mp_* (softening of plasticizing additive).

Cycle	Sample	*T_g_^SS^* [°C]	*T_g_^HS^* [°C]	*T_mp_* [°C]
1st heat	A	−38.1	63.2	211.5
2nd heat	A	−30.2	112.2	-
1st heat	B	−37.4	65.8	208.3
2nd heat	B	−33.9	108.1	-

**Table 6:** Specific heat analysis results.

Sample	Specific Heat *C_p_* [J/g⸳K]							
	*T_g_* [°C]	20 °C	40 °C	60 °C	80 °C	100 °C	120 °C	140 °C
A	1.843	1.728	1.812	1.874	1.951	2.022	2.100	2.170
B	1.470	1.292	1.414	1.470	1.527	1.583	1.640	1.694
Difference	–	0.436	0.398	0.404	0.424	0.439	0.460	0.476

## Data Availability

Not applicable.
